# Efficacy and Safety of Dulaglutide Compared to Liraglutide: A Systematic Review and Meta-analysis in Patients with Type 2 Diabetes Mellitus

**DOI:** 10.22037/ijpr.2019.14733.12619

**Published:** 2019

**Authors:** Saeed Taheri, Ali Saffaei, Bahman Amani, Arash Akbarzadeh, Farzad Peiravian, Nazila Yousefi

**Affiliations:** a *Department of Pharmacoeconomics and Pharmaceutical Management, School of Pharmacy, Shahid Beheshti University of Medical Sciences, Tehran, Iran. *; b *Student Research Committee, Department of Clinical Pharmacy, School of Pharmacy, Shahid Beheshti University of Medical Sciences, Tehran, Iran. *; c *Department of Health Sciences Education Development, School of Public Health, Tehran University of Medical Sciences, Tehran, Iran. *; d *Department of Epidemiology and Biostatistics, School of Public Health, Tehran University of Medical Sciences, Tehran, Iran.*

**Keywords:** Diabetes Miletus, Dulaglutide, Liraglutide, Meta-analysis, Systematic review

## Abstract

Diabetes mellitus has been always one of the most prevalent chronic diseases in the last decades. There exist a wide range of pharmacological agents for controlling this disease. However, these agents fare differently in terms of efficacy and safety. Hence, the aim of this study was to compare dulaglutide and liraglutide, two glucagon-like peptide-1 receptor agonists, in terms of efficacy and safety, drawing on a systematic review and meta-analysis. A systematic review and meta-analysis were carried out in January 2018. The articles were evaluated by two independent investigators and their quality was evaluated using Jadad scale and the Cochrane Collaboration’s tools. Finally, the eligible articles entered the study. HbA1c and FBS were considered as efficacy outcomes. Safety profile was evaluated based on several outcomes such as serious side effects and vital signs. Three articles met the inclusion and exclusion criteria. The results indicated that the mean difference (MD) of HbA1c reduction was -0.10% (95% CI, -0.20% to -0.01%, P=0.03) in the patients who received dulaglutide in comparison with the patients who received liraglutide. In addition, dulaglutide was safer than liraglutide in terms of gastrointestinal problems (RR=0.85, 95% CI, 0.73 to 0.99, P=0.04, I^2^=55%) and heart rate (RR=-1.14, 95% CI, -1.90 to -0.38, P=0.003, I^2^=0%). Once-weekly dulaglutide showed a further reduction in HbA1c compared to once-daily liraglutide. However, comparisons between these regimens indicated no significant difference between groups in either FBS reduction or safety profile. Similarly, no statistically significant difference was observed in treatment discontinuation, hypoglycemia events, and vital signs.

## Introduction

Diabetes mellitus (DM), a chronic metabolic disease, may impair insulin secretion, insulin action, or both. This condition leads to hyperglycemia and micro vascular problems (1). The prevalence of DM has been growing rapidly in the last couple of years. Obesity, ageing population, and lack of physical activities are mainly to blame for this rapid progression (2). DM categorized to type 1 DM and type 2 DM has been branded as ‹Black Death′ in recent years (3). Efficient glycemic control plays essential crucial role in regulating blood glucose balance and preventing micro vascular complications (4, 5). Epidemiological studies reveal that near 90% of DM patients are diagnosed with type 2. Early pharmacological intervention following DM diagnosis has been recommended strongly, and metformin is considered as the drug of choice for the first line treatment. Nevertheless, metformin has failed to change the progressive nature of type 2 DM (6, 7). Several trials have demonstrated that the combination therapy has been more efficacious and is better tolerated than high doses of each anti-diabetic agents (8). In this regard, a recent statement from American Diabetes Association (ADA) and European Association for Study of Diabetes (EASD) recommends combinational therapy for the patients with hemoglobin A1c (HbA1c) > 9%. However, the conventional therapeutic interventions for type 2 DM cannot control hyperglycemia effectively. In addition, several side effects of these agents reduce the patient′s adherence to their medications (9). Hence, it seems necessary to investigate the new pharmacological agents for improving the management of this disease more thoroughly. Recently, new pharmacological agents, i.e. Glucagon-like peptide-1 receptor agonists (GLP-1 RAs), have played a considerable role in the treatment of diabetes mellitus (10). GLP-1 RAs are injectable peptides which are structurally and functionally similar to endogenous incretin GLP-1 whose secretion failure has a critical pathophysiologic role in DM (11). However, as they are not neutralized by the dipeptidyl peptidase-4 (DPP-4), their half-life is longer than that of endogenous GLP-1 (12). GLP-1 RAs are categorized as short-acting (exenatide and lixisenatide), and long-acting agents (liraglutide, dulaglutide, albiglutide, and semaglutide) (13). Although all these medications potently decrease HbA1c, some fundamental differences arise among them when such factors as fasting and postprandial hyperglycemia reduction, potency of weight loss, cardiovascular protection efficacy, and adverse events profile are brought to bear (14, 15). In addition, dulaglutide is processing to be entered in Iran drug list and may be in the Iran market in near future. Against this backdrop, the aim of the present study was to compare dulaglutide and liraglutide in terms of efficacy and safety, drawing on a systematic review and meta-analysis.

## Methods

The Preferred Reporting Items for Systematic Reviews and Meta-Analyses (PRISMA) guideline was used to carry out the systematic review (16).

To evaluate the efficacy and safety of dulaglutide compared to liraglutide, a systematic review and meta-analysis was carried out. 

*Literature search strategy*

To begin with, appropriate keywords were selected based on MeSh terms and a comprehensive review was done in January 2018. The electronic databases including PubMed, Embase, Scopus, Cochrane library, and Web of science as well as non-electronic databases such as library literature were investigated. In addition, Open Gray database, EU CTR database, Google Scholar, and ClinicalTrials.gov database were explored to find unpublished records and conference proceedings. To ensure a more sensitive search, all searches were done based on the two main keywords, i.e. dulaglutide and liraglutide (or their brand names). No limitations such as year of publication or language were set in this conducting search. 

*Inclusion and Exclusion Criteria*

To find eligible articles, the following inclusion and exclusion criteria were applied. The inclusion criteria were (1) study population: patients with type 2 DM; (2) intervention: dulaglutide administration with or without a metformin background therapy; (3) comparison: liraglutide administration with or without a metformin background therapy; (4) efficacy outcome: HbA1C and fasting serum glucose (FSG); (4) safety outcomes: nausea, diarrhea, constipation, gastrointestinal disorders, incidence of serious adverse effects, discontinuation of study, or vital signs; and (5) study design: clinical trials. 

The exclusion criteria were: (1) study population: patients with other types of diabetes except type 2 DM; (2) intervention: dulaglutide administration in combination with other antidiabetic agents; (3) comparison: liraglutide administration in combination with other anti-diabetic agents; (4) outcomes: studies assessing irrelevant outcomes; and (5) study design: studies designed inappropriately, studies in which suffering from evident biases and other studies except clinical trial. In addition, studies in outpatient settings were left out of this study.

*Study selection and appraisal*

Then, the selected articles were transferred to the Mendeley reference manager software and duplicated documents found in different databases were counted out. Then, the full texts of the articles were screened by two reviewers ensure compliance with the inclusion and exclusion criteria and, finally, the data from eligible studies were extracted onto an Excel spreadsheet. In case of disagreement between researchers, a third reviewer was decided on discrepancies of the articles. 

The authors independently used the modified Jadad scale to assess the methodological quality of each included study. If two authors had different opinions when assessing and selecting the studies, the consensus was reached by the intervention of a third party. The modified Jadad scale includes 8 items to evaluate as follows: if randomization was done (score range 0–1); if randomization was appropriate (score range −1 to 1); if blinding was done (score range 0–1); if blinding was appropriate (score range −1 to 1); if withdrawals and dropouts were described (score range 0–1); if inclusion and exclusion criteria were described (score range 0–1); if adverse reactions were assessed (score range 0–1); and if the statistical analysis was described (score range 0–1) (17). The score of each study ranges from 0 (the lowest quality) to 8 (the highest quality). Studies were classified as moderate if they had a score of 4 or 5. All included trials were also evaluated using Cochrane Collaboration’s tool for assessing the risk of bias in randomized trials and the Grading of Recommendations Assessment, Development and Evaluation Working Group grading scheme (18, 19). 

*Data analysis*

The data were analyzed using the mean difference (MD) with 95% confidence intervals (95% CIs). RevMan version 5.3.5 software (The Nordic Cochrane Center, The Cochrane Collaboration, Copenhagen, Denmark) was used for all data analyses. Meta-analysis was conducted when the trials had an acceptable clinical homogeneity and statistical heterogeneity limit. Heterogeneity was quantified using the Cochran Q test and I^2^ statistics. A *P* value < 0.10 for Chi-square testing of the Q statistic or an I^2^ > 30% was considered as a statistically significant heterogeneity that leads to use random effect and otherwise fixed effect in accordance with the Cochrane methodology (20, 21).

## Results

*Study characteristics*

**Figure 1:**
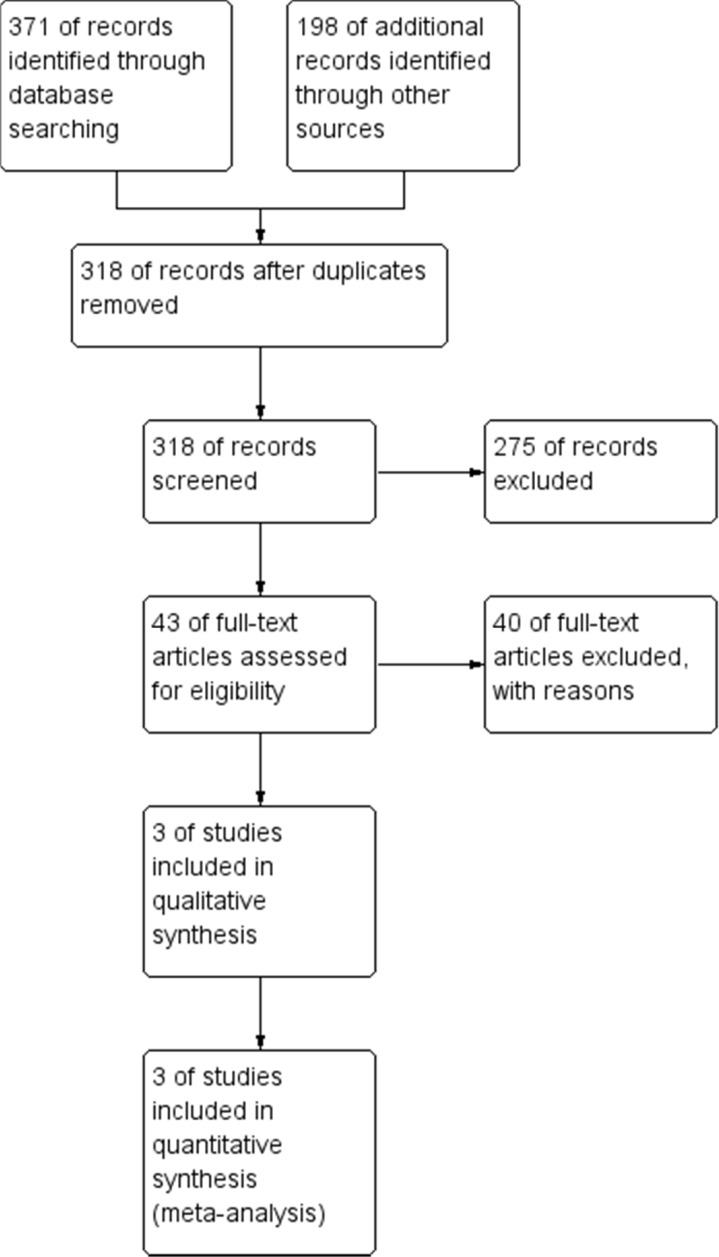
The PRISMA flow diagram of the study

In this comprehensive systematic review and meta-analysis, some databases were searched with appropriate keywords and, overall, 318 articles were included in the study. After primary evaluation by screening the titles and abstracts, according to the inclusion criteria, 43 articles were selected for full-text evaluation. Finally, three articles were selected for final analysis whose characteristics have been presented in Table 1 (22–24). The PRISMA flow chart of this study is given in Figure 1.

**Figure 2:**
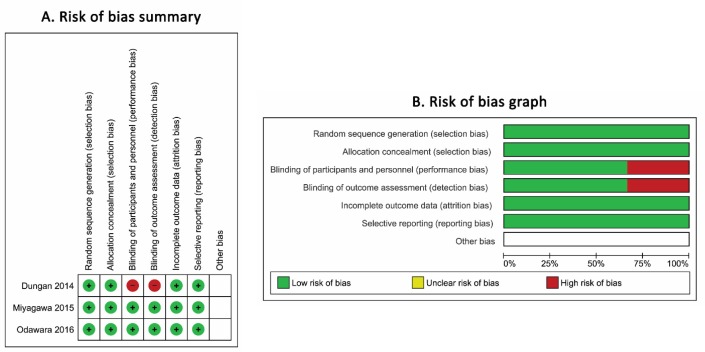
Risk of bias assessment by the Cochrane Collaboration’s tool

The Jadad score of the selected articles showed that 2 of the included studies have a moderate quality. However, the result of the Cochrane Collaboration’s tool for assessing risk of bias in included randomized trials shows a low bias as illustrated in Figure 2.

*Efficacy*

**Figure 3:**
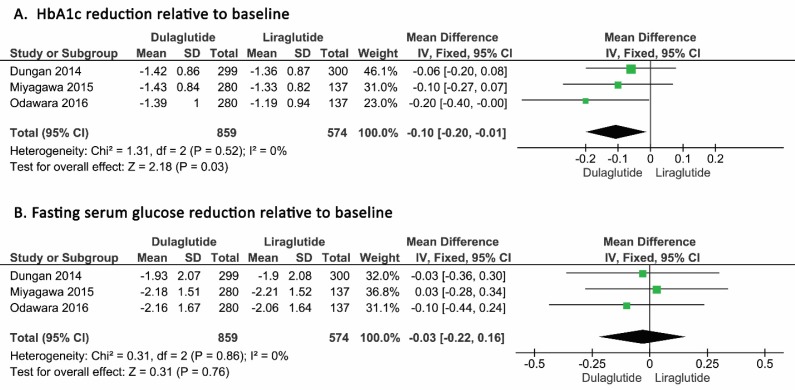
Pooled mean difference of HbA1c reduction (A) and FSG reduction (B) in patients who received dulaglutide compared to liraglutide

In terms of HbA1C, as heterogeneity among the selected studies based on I^2^ = 0 was not seen, a fixed model was developed for analysis. After analyzing the 1433 patients, the mean differences of HbA1c reduction was found to be -0.10% (95% CI, -0.20% to -0.01%, P=0.03) in patients who received dulaglutide in comparison with patients who received liraglutide. As shown in the forest plot, this reduction was statistically significant (Figure 3, Panel A).

In terms of FSG, heterogeneity was not seen among the selected studies based on I^2^ = 0 and a fixed model was developed for analysis. 

The mean difference of FSG was 0.03 mmol/L (95% CI, -0.18 mmol/L to -0.24 mmol/L, P=0.76) in the patients who received dulaglutide in comparison with the patients who received liraglutide, and this reduction was not significant as shown in the relevant forest plot (Figure 3, Panel B).

*Safety*

**Figure 4:**
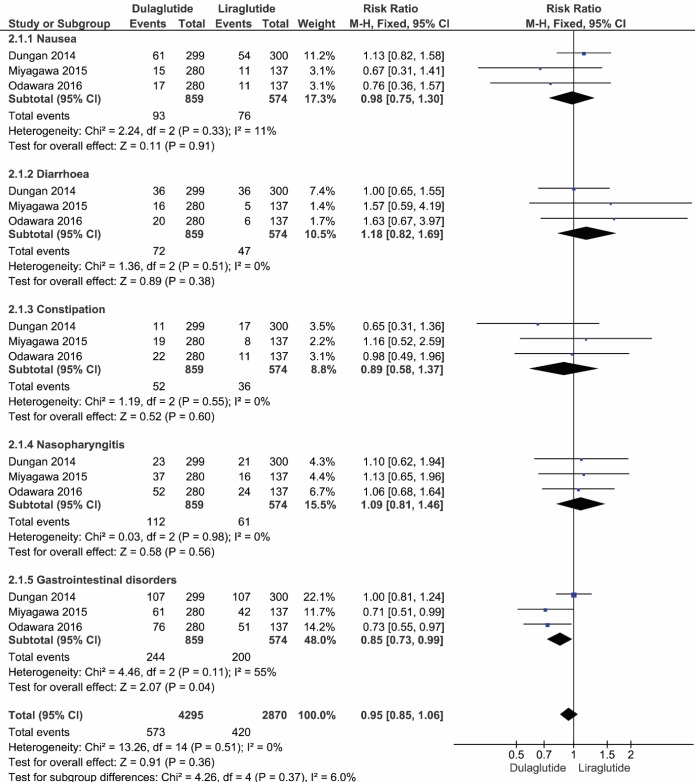
The forest plot showing the incidence of adverse events in patients who received dulaglutide compared to liraglutide

Based on the I^2^ score, the fixed model was applied to analyze the safety of dulaglutide and liraglutide. The results of 1433 patients showed that the adjusted risk ratio for the incidence of nausea was 0.98 (95% CI, 0.75 to 1.3, P=0.91, I^2^=11%), which was not statistically significant. Similarly, the adjusted risk ratio for the incidence of diarrhea was not significantly different between these two agents, 1.18 (95% CI, 0.82 to 1.69, P=0.38, I^2^=0%). Likewise, the adjusted risk ratio of the incidence of constipation, 0.89 (95% CI, 0.58 to 1.37, P = 0.60, I^2^=0%), and adjusted risk ratio of the incidence of nasopharyngitis, 1.09 (95% CI, 0.81 to 1.46, P=0.56, I^2^=0%) were not statistically different. However, the adjusted risk ratio of the incidence of gastrointestinal was found to be statistically significant between dulaglutide and liraglutide (RR: 0.85, 95% CI, 0.73 to 0.99, P=0.04, I^2^=55%). Finally, the pooled data for the incidence of total adverse events showed no significant difference (RR: 0.95, 95% CI, 0.85 to 1.06, P=0.51, I^2^=0%) (Figure 4).

*Serious adverse events*

**Figure 5:**
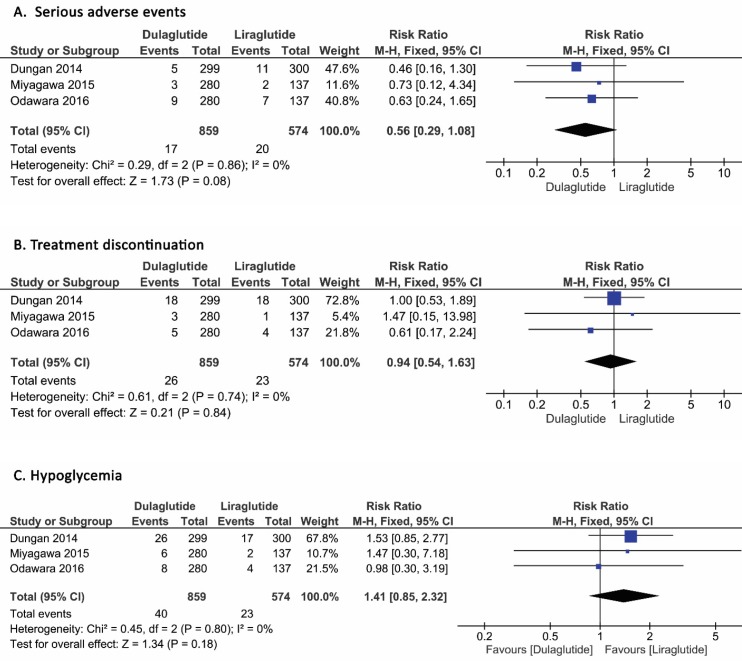
Incidence of serious adverse events (A), Treatment discontinuation (B), and Hypoglycemia (C) in patients who received dulaglutide compared to liraglutide

The adjusted risk ratio of serious adverse events in 1433 patients on the fixed model was 0.56 (95% CI, 0.29 to 1.08, P=0.08, I^2^=0%), which, as can be seen in the forest plot, was not different between dulaglutide and liraglutide (Figure 5, Panel A).

*Discontinuation of Treatment*

The discontinuation of treatment was similar between the patients who received dulaglutide and liraglutide. 

The adjusted risk ratio of discontinuation of treatment in 1433 patients was 0.94 (95% CI, 0.54 to 1.63, P=0.84, I^2^=0%) (Figure 5, Panel B).

*Hypoglycemia*

The adjusted risk ratio of different types of hypoglycemia events in 1433 patients was 1.41 (95% CI, 0.85 to 2.32, P=0.80, I^2^=0%), which was not different between dulaglutide and liraglutide, based on the fixed model (Figure 5, Panel C).

*Vital Signs*

Due to heterogeneity among the selected studies regarding systolic blood pressure (I^2^=73%), the random model was utilized to analyze the mean difference of vital signs between dulaglutide and liraglutide. Among vital signs, the mean differences of the heart rate was -1.14 (95% CI, -1.90 to -0.38, P=0.003, I^2^=0%) in favor of dulaglutide. However, other vital signs, such as systolic blood pressure and diastolic blood pressure, were not significantly different between the two medications 

(Figure 6).

**Figure 6:**
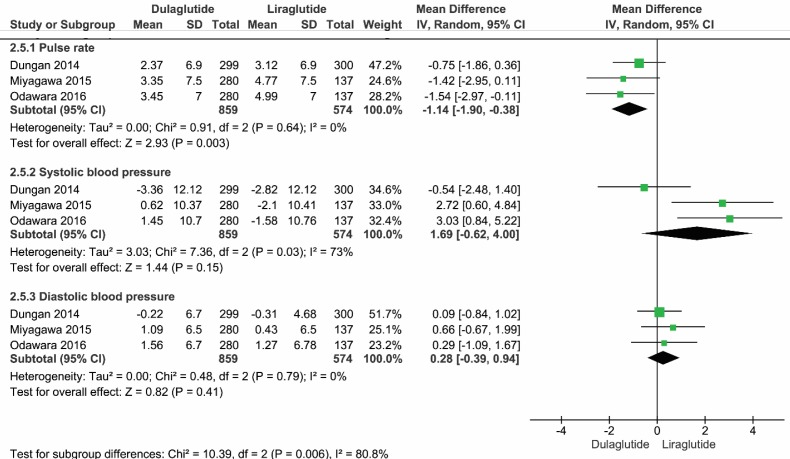
Forest plot showing the mean difference of vital signs in patients who received dulaglutide compared to liraglutide

**Table 1:** Study characteristics

**Authors**	**Year**	**Country**	**Design**	**Duration (Week)**	**Sample Size (DLG:LRG)**	**Doses (DLG:LRG)** **in mg**	**Jadad Score**
Odawara(23)	2016	Japan	Parallel	52	280:137	0.75:0.9	4
Miyagawa(22)	2015	Japan	Parallel	26	280:137	0.75:0.9	4
Dungan(24)	2014	International	Parallel	26	299:300	1.5:(0.6-1.8)	3

## Discussion

To the best of the authors′ knowledge, this study is the first systematic review and meta-analysis aiming to compare two long-acting GLP-1 receptor agonists, i.e. dulaglutide and liraglutide, which are prescribed to subjects with type 2 diabetes with or without other hypoglycemic drugs. 

The systematic review identified 3 trials reporting results for 26 to 52 weeks (22–24). Based on pooled estimates from 3 included RCTs, once-weekly dulaglutide is associated with more reduction in HbA1c than once-daily liraglutide. This difference between weekly and daily GLP-1 RAs may be attributed to the potential impact of the weekly agents on both FBS and postprandial plasma glucose (PPG), compared to the daily agents that may predominantly regulate PPG (10). 

However, there were no statistically significant differences in the FBS reduction outcome. Although FBS is an important indicator of DM management, this indicator reflects the short-term efficacy. Indeed, HbA1c has more reproducibility compared to FBS, reflecting the glucose control condition for the past three months. HbA1c is also identified as a superior predictive factor for diabetic retinopathy and cardiovascular related problems (25, 26). It inevitably follows that dulaglutide has a higher long-term efficacy in glucose management compared to liraglutide.

In terms of safety profile, the results indicated that there were no weight-sparing benefits for either agent, while only dulaglutide was associated with a lower gastrointestinal complications and as well as a reduction in heart rate. Regarding the lower gastrointestinal problems with dulaglutide compared to liraglutide, the most probable mechanism for this phenomenon is the delayed gastric emptying since liraglutide reduces duodenal and small intestine motility (27). The previous randomized trials have revealed that in obese patients with cardiovascular diseases, liraglutide increased the heart rate despite a significant weight reduction and improvement in metabolic profile (28). 

The risk of hypoglycemia, which can prove a daunting challenge and obstacle for the treatment of diabetes, was also similar between dulaglutide and liraglutide. However, results should interpret with caution since due to the variability in definitions of hypoglycemia in different studies, a challenge has been identified by the American Diabetes Association (8). No statistically significant difference was observed in treatment of the discontinuation and other vital signs.

Although some systematic reviews, meta-analyses, and network meta-analyses of once-weekly GLP-1 RAs had been previously published (10,29–39), the research question of the current study was more specific than that posed by previous studies. In our analyses, only head-to-head comparisons between dulaglutide and liraglutide have been considered, focusing on the HbA1c reduction and FBS reduction outcomes, which offers more comprehensive evidence for clinicians regarding choice between the two injectable options in the management of type 2 diabetes. 

In addition, none of the previously published analyses compared once-weekly dulaglutide with once-daily liraglutide in terms of safety and efficacy except one which has compared dulaglutide and liraglutide indirectly as a part of clinical trial planning (32). Some of the previously published analyses compared GLP-1 RAs with placebo or basal insulin (10, 33). The other available studies assessed the micro vascular effects or cardiovascular events of GLP-1 RAs without attention to HbA1c reduction (36, 37, 39).

*Limitation*

Limitations regarding the body of evidence and the review process should be taken into account when interpreting our findings. First of all, the number of studies included in this meta-analysis is small; however, we thoroughly searched relevant databases and ensured that no study had been left out. 

Second, all included studies received funding from pharmaceutical companies which could skew the results in their favor. Third, as the trials followed the patients up to 52 weeks, the conclusions drawn about the long-term efficacy and safety should not be taken at face value, and the other relevant factors should also be brought to bear on our interpretation of the findings. Fourth, the result of hypoglycemia incidence should be interpreted with caution due to a high heterogeneity in defining hypoglycemic events. Fifth, long-term observational studies are needed to draw safer inferences regarding the safety profile, i.e. rare events, of once-weekly GLP-1 RAs, such as pancreatitis, cardiovascular outcomes, and cancer (40). Sixth, the generalizability of our results is attenuated to conduct sub-group analysis for different populations, such as elderly patients or patients with cardiovascular comorbidities or with a chronic renal disease. 

Finally, patient-important outcomes, such as quality-adjusted life years, which can be important determinants of health-related quality of life in the patients with type 2 diabetes, were not assessed in our analysis (41). 

It seems that the once-weekly dulaglutide should be investigated in terms of cost-effectiveness compared to the once-daily liraglutide to provide stronger evidences for decision-makers to allocate healthcare resources properly.

## Conclusion

The current analysis reveals that once-weekly dulaglutide has a greater reduction in HbA1c compared to once-daily liraglutide. However, the comparison between these regimens indicated no significant difference in FBS changes relative to the baseline. 

In addition, there were no significant differences in adverse events for either agent, while only dulaglutide was associated with a lower gastrointestinal complication and also a reduction in heart rate. Further studies are needed to make future meta-analysis more robust in terms of the safety and efficacy of these drugs.
